# CAR-T cell therapy clinical trials: global progress, challenges, and future directions from ClinicalTrials.gov insights

**DOI:** 10.3389/fimmu.2025.1583116

**Published:** 2025-05-20

**Authors:** Li-Ya Cao, Yue Zhao, Yang Chen, Pan Ma, Jiang-Chuan Xie, Xin-Mei Pan, Xin Zhang, Yong-Chuan Chen, Qian Wang, Lin-Li Xie

**Affiliations:** ^1^ Department of Pharmacy, the First Affiliated Hospital of Army Medical University (Third Military Medical University), Chongqing, China; ^2^ Pharmacy Department, Chongqing Emergency Medical Center, Chongqing University Central Hospital, Chongqing, China

**Keywords:** chimeric antigen receptor T-cell, clinical trials, clinicaltrials.gov, hematological malignancies, solid tumors, publication following search queries: condition/disease or intervention/treatment: “CAR T”, “chimeric antigen receptor”, “CAR T cell”

## Abstract

Chimeric antigen receptor T (CAR-T) cell therapy has undergone vigorous development in recent years, yet it still faces significant challenges and difficulties in its clinical application and further development. A systematic synthesis of global trends in CAR-T clinical trials is essential to identify knowledge gaps, optimize treatment strategies, and guide future research directions. This review analyzed 1,580 CAR-T clinical trials registered at ClinicalTrials.gov as of April 2024, and extracted characteristic data in multiple dimensions, including target specificity, treatment indication, and development stage etc. The transparency of trial outcomes was assessed by validation with articles published in PubMed/Google Scholar. Additionally, it is complemented by investigator surveys assessing to barriers to CAR-T development, prospects, and recommendations.

## 1 Introduction

Chimeric antigen receptor T (CAR-T) cell therapy is a groundbreaking immunotherapy that confers target-specific recognition capacity to autologous or allogeneic T cells through genetic engineering. The canonical CAR architecture consists of three essential components: an extracellular antigen-binding single-chain variable fragment (scFv), a transmembrane domain, and intracellular activation/co-stimulatory signaling domains (e.g., CD28, 4-1BB). This unique design enables direct binding to target antigens via a major histocompatibility complex (MHC)-independent mechanism, triggering T cell cytotoxic activity and thus representing one of the most promising therapeutic strategies in modern medicine, especially in oncology (1). CAR-T therapy has achieved remarkable success in hematologic malignancies, with anti-CD19 products revolutionizing B-cell malignancy management and constituting >50% of investigational or commercialized cell therapies (2, 3). Emerging applications now extend to solid tumors, autoimmune disorders, and infectious diseases (4–6). However, critical scientific and translational barriers hinder its broader clinical translation. In hematological contexts, the long-term efficacy is still limited by insufficient expansion and poor CAR-T cell persistence (7). Solid tumors present amplified challenges: the immunosuppressive tumor microenvironment (TME) impedes therapeutic success through physical barriers, metabolic competition, and immune checkpoint overexpression, collectively suppressing T cell infiltration, survival, and effector functions while promoting exhaustion (8). Antigen escape mechanisms – notably target loss (e.g., CD19-negative relapse in 30–50% of B-ALL cases) or downregulation – further drive therapeutic resistance (9, 10). In addition, the development of optimally effective CAR-T cell therapies necessitates a balanced integration of potency and safety, particularly addressing the two most critical toxicities: cytokine release syndrome (CRS) and immune effector cell-associated neurotoxicity syndrome (ICANS). These toxicities are mechanistically intertwined with CAR-T cell activation kinetics and tumor burden, necessitating risk-adapted designs (8). Additional accessibility barriers arise from complex manufacturing processes and exorbitant costs (>$500,000 per treatment course), compounded by developmental attrition rates where only 35% of initiated trials progress beyond Phase 2 (11).

ClinicalTrials.gov, the largest publicly accessible online database, reveals a rapidly evolving landscape (11). This review synthesizes global trends from 1,580 CAR-T trials registered on ClinicalTrials.gov (2003-2024), to map the global trajectory of CAR-T development, dissect unresolved challenges, and propose actionable strategies to accelerate clinical translation. Based on the publicly available contact information, we surveyed the CAR-T researchers to identify barriers to research advancement and result dissemination, and to propose collaborative strategies.

## 2 Data landscape and methodological framework

### 2.1 Retrieval and screen of relevant registered trials

In April 2024, we retrieved data from ClinicalTrials.gov to identify relevant CAR-T studies. To ensure comprehensive coverage, the research team employed the following search queries: Condition/disease or Intervention/treatment: “CAR T”, “Chimeric Antigen Receptor”, “CAR T cell”, “CAR T cell Therapy”, “CAR T Immunotherapy” “CAR-T Therapy”, “Chimeric Antigen Receptor T-Cell Immunotherapy”, “Chimeric Antigen Receptor T-cell Therapy”, “Chimeric Antigen Receptor T-cell”. No restrictions were placed on study status. Due to the similarity of search terms, deduplication was performed to eliminate redundant entries. Subsequently, a manual review of the registration information for all entries was conducted to exclude studies unrelated to CAR-T. The studies included in this analysis featured CAR-T cells as either the sole or combined intervention.

### 2.2 Data extraction, validation and researcher perspectives

We conducted a comprehensive search for information on various aspects of CAR-T studies for disease treatment, including targets, CAR-T generation, costimulatory domains, transfection methods, cell sources, disease and specific conditions treated, intervention strategies, sponsors/collaborators and funding institutions, phases, status, start dates, and locations.

To comprehensively evaluate the disclosure of results from CAR-T studies, we matched ClinicalTrials.gov entries (NCT identifiers) with published studies on PubMed and Google Scholar. Validation criteria included study design, indication, primary purpose, and principal investigator.

In order to thoroughly assess the status and challenges of completed CAR-T studies (including those in completed, terminated, and withdrawn states), the project team conducted a web-based survey utilizing the email addresses of research contacts published on ClinicalTrials.gov. Non-response was defined as the absence of a reply to two consecutive emails within a 14-day period. Cases where no email address was provided, the email address could not be located, or the email address was invalid were categorized as “unable to contact”. The survey content encompassed research obstacles, reasons for discontinuation, publication status and intentions, and suggestions for CAR-T research strategies, among other topics. The specific survey instrument is provided in the appendix.

### 2.3 Analytical approach

The characteristics of the trials were delineated in accordance with the information available on ClinicalTrials.gov. Categorical data were presented as frequencies and percentages. Comparative analyses of categorical variables across groups were conducted utilizing the chi-square test. P-value of < 0.001 was deemed to indicate statistical significance. For the survey component, responses were scrutinized and summarized through frequency and percentage metrics. All statistical analyses were executed using SPSS 25.0 software (IBM Corp., Armonk, NY, USA).

## 3 Global landscape of CAR-T clinical trials

**Figure 1:**
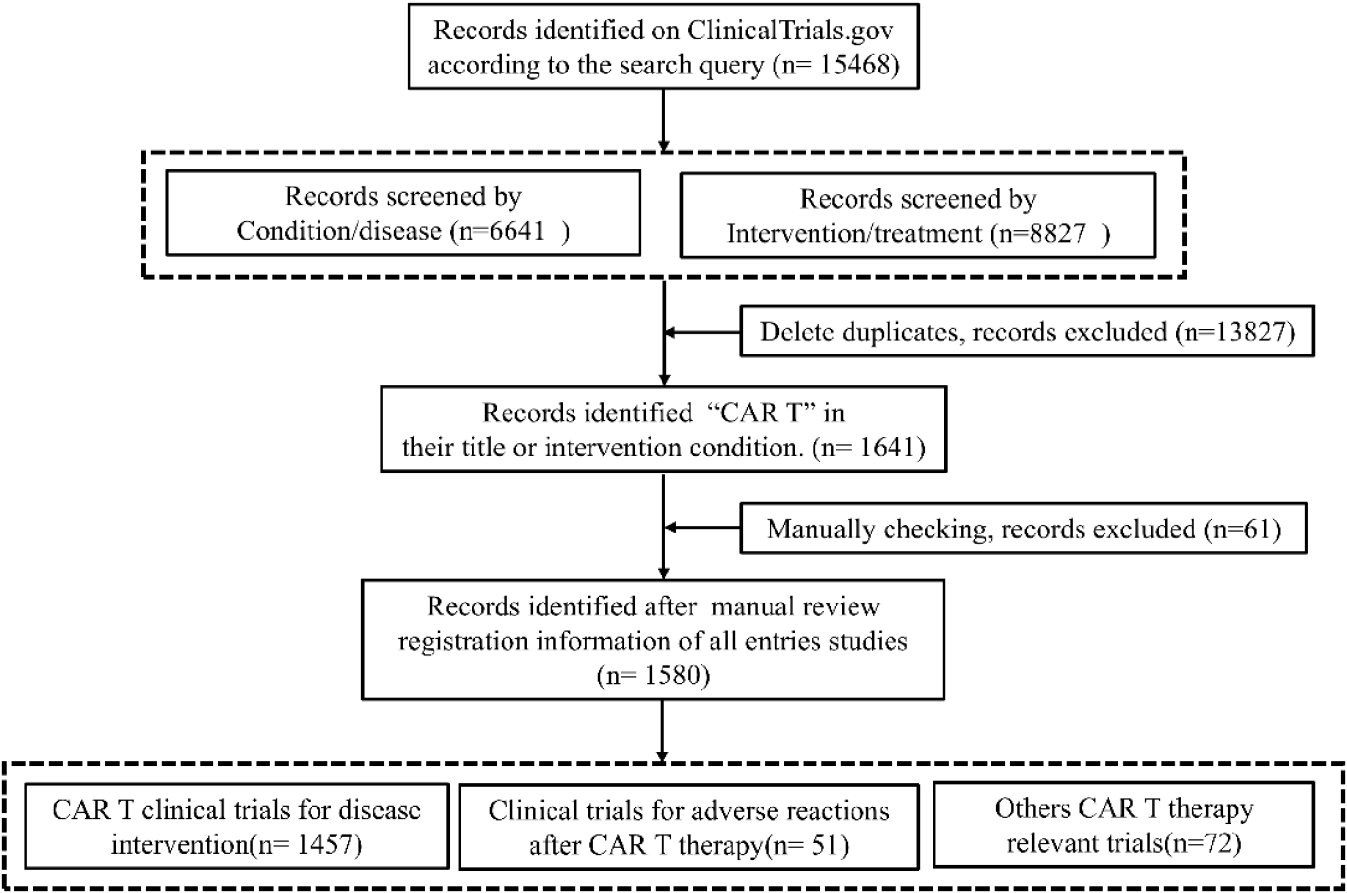
CAR-T clinical trials screening flow diagram.

Systematic analysis of ClinicalTrials.gov records identified 1,641 CAR-T-related entries, with 1,580 trials meeting eligibility after deduplication and manual screening (**Figure 1**). The topics of CAR-T Clinical Trials spanned several domains: primary interventions, Toxicity management and Health economics & biomarkers. 1,457 trials (92.2%) evaluated CAR-T as monotherapy or combined regimens across hematologic (71.6%), solid (24.6%), and autoimmune malignancies (2.75%). 51 trials (3.2%) focused on mitigating adverse events like CRS. Remaining studies (4.6%) addressed cost-effectiveness, quality-of-life metrics, and predictive biomarkers.

### 3.1 Growth trends and geographical distribution

**Figure 2:**
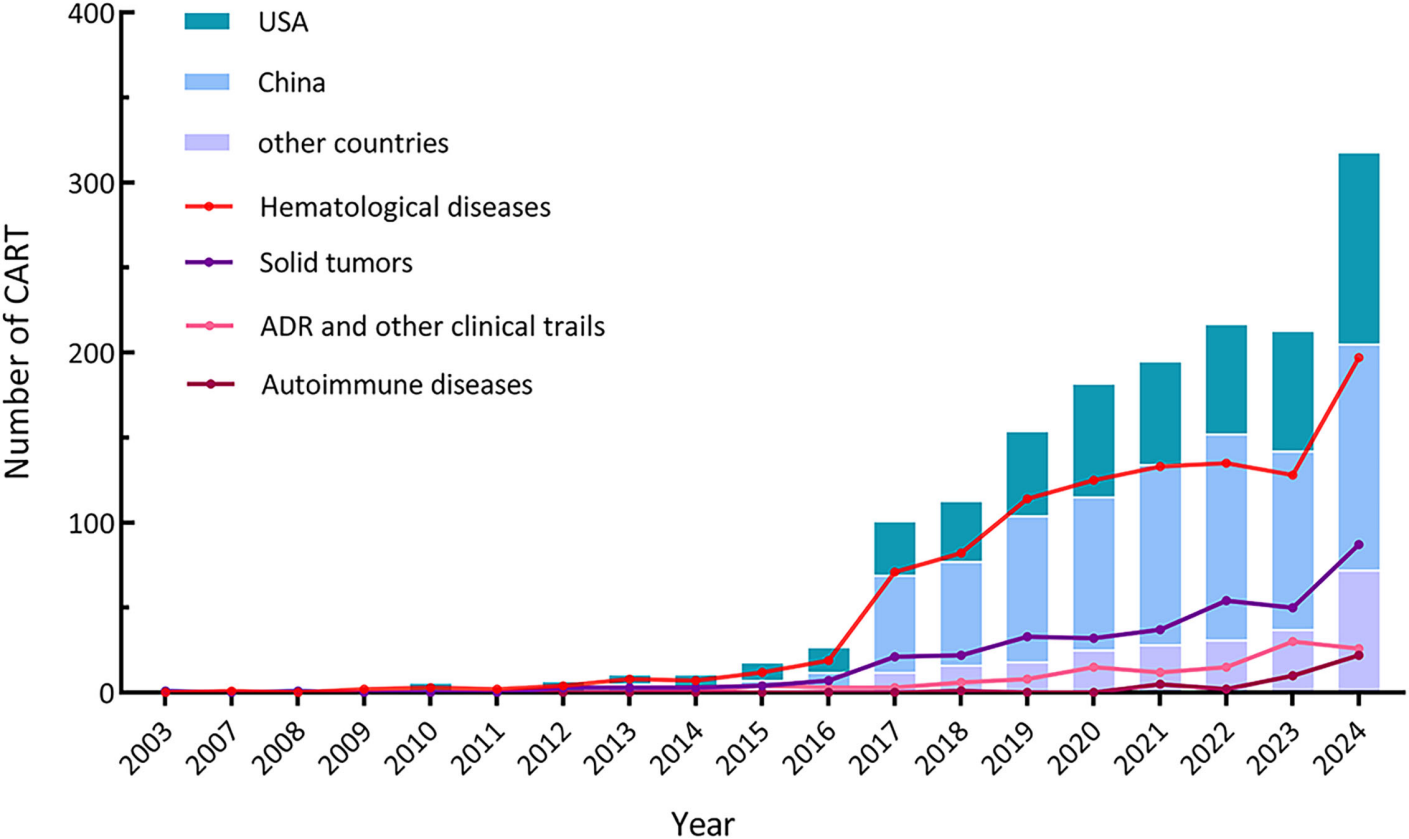
Distribution of CAR-T trials conducted country, indication, and year.

The earliest CAR-T study registered on ClinicalTrials.gov dates back to 2003, and the number of CAR-T studies carried out worldwide showed an overall trend of increasing year by year, and entered a rapid growth in 2017. It is worth mentioning that China has been in a leading position in the number of CAR-T studies, but the registered quantity decreased from 2022 to 2023. Other CAR-T studies have mainly come from the United States with a steady upward trend. The increase mainly camas from the hematological diseases and solid tumors, and both increased rapidly in 2017 (**Figure 2**). In 2021, the number of clinical trials registered for CAR-T studies in autoimmune diseases (such as refractory systemic lupus erythematosus) began to increase.

In recent years, clinical trials specifically focusing on adverse reactions associated with CAR-T therapy have emerged. Overall, despite the significant increase in the quantity of clinical trials, the majority of registered trials are in the early phases with limited participant enrollment. Among the registered CAR-T clinical trials, only 170 were Phase 2, Phase 3, or Phase 4 trials, while 891 were categorized Phase 1 or early Phase 1 trials.

### 3.2 Sponsor/collaborators and funding

Sponsors and collaborators have provided essential support for CAR-T research, including funding, design, implementation, and data analysis. The majority of registered studies are funded by non-profit organizations or local academic institutions (**Table 1**). These sponsors primarily account for nearly 50% of the CAR-T clinical trials in China and approximately 40% of trials in other regions, with industry-funded studies constituting the second-largest category. Globally, over 30% of the trials have a mixed funding source, involving collaborations between industry, the National Institutes of Health (NIH), academic institutions, and non-profit organizations. Notably, approximately 40% of the registered CAR-T clinical trials in China are funded by mixed sources, compared to about 24% in other regions. The involvement of the NIH and U.S. federal agencies introduces additional differences in funding sources between China and the United States. The top 20 institutions and pharmaceutical industry ranked by the number of clinical trials sponsored are shown in **Table 2**. Most of the trials funded by these agencies are collaborative. It is worth mentioning that the top five sponsors or collaborators of the most trials were all non-profit organizations, and there are only 6 pharmaceutical companies among the 20 centers. Although pharmaceutical industry participation remains limited overall, its proportion has increased compared to 2021 levels.

**Table 1:** Funding source of registered clinical trials.

Funding source	Global	China	Other regions
Other	687	352	335
Industry|Other	437	313	124
Industry	356	115	241
NIH|Other	51		51
NIH	29		29
Industry|Other|NIH	10		10
Other|Industry|U.S. Fed	3		3
Other|U.S. Fed	3		3
Industry|NIH	1		1
Other|NIH|U.S. Fed	1		1

**Table 2:** The top 20 institutions and pharmaceutical industry ranking by the number of clinical trials sponsored.

Rank	Sponsor/Collaborators	Individual (n)	cooperative (n)	Total (n)
1	National Cancer Institute (NCI)	3	82	85
2	Zhejiang University	18	50	68
3	Baylor College of Medicine	6	34	40
4	Chinese PLA General Hospital	29	12	41
5	University of Pennsylvania	25	17	42
6	Shenzhen Geno-Immune Medical Institute	24	11	35
7	Yake Biotechnology Ltd.	0	35	35
8	Gilead Sciences(Kite, A Gilead Company|Gilead Sciences)	21	16	37
9	Chongqing Precision Biotech Co., Ltd	24	10	34
10	Hebei Senlang Biotechnology I	17	16	33
11	The First Affiliated Hospital of Soochow University	10	23	33
12	PersonGen BioTherapeutics (Suzhou) Co., Ltd.	4	28	32
13	City of Hope Medical Center	1	28	29
14	Memorial Sloan Kettering Cancer Center	15	14	29
15	National Institutes of Health Clinical Center (CC)	0	26	26
16	Novartis	16	8	24
17	UNC Lineberger Comprehensive Cancer Center	8	14	22
18	Hebei Yanda Ludaopei Hospital	2	18	20
19	Wuhan Union Hospital, China	1	19	20
20	Institute of Hematology & Blood Diseases Hospital, China	5	13	18

### 3.3 Indications and targets

**Figure 3:**
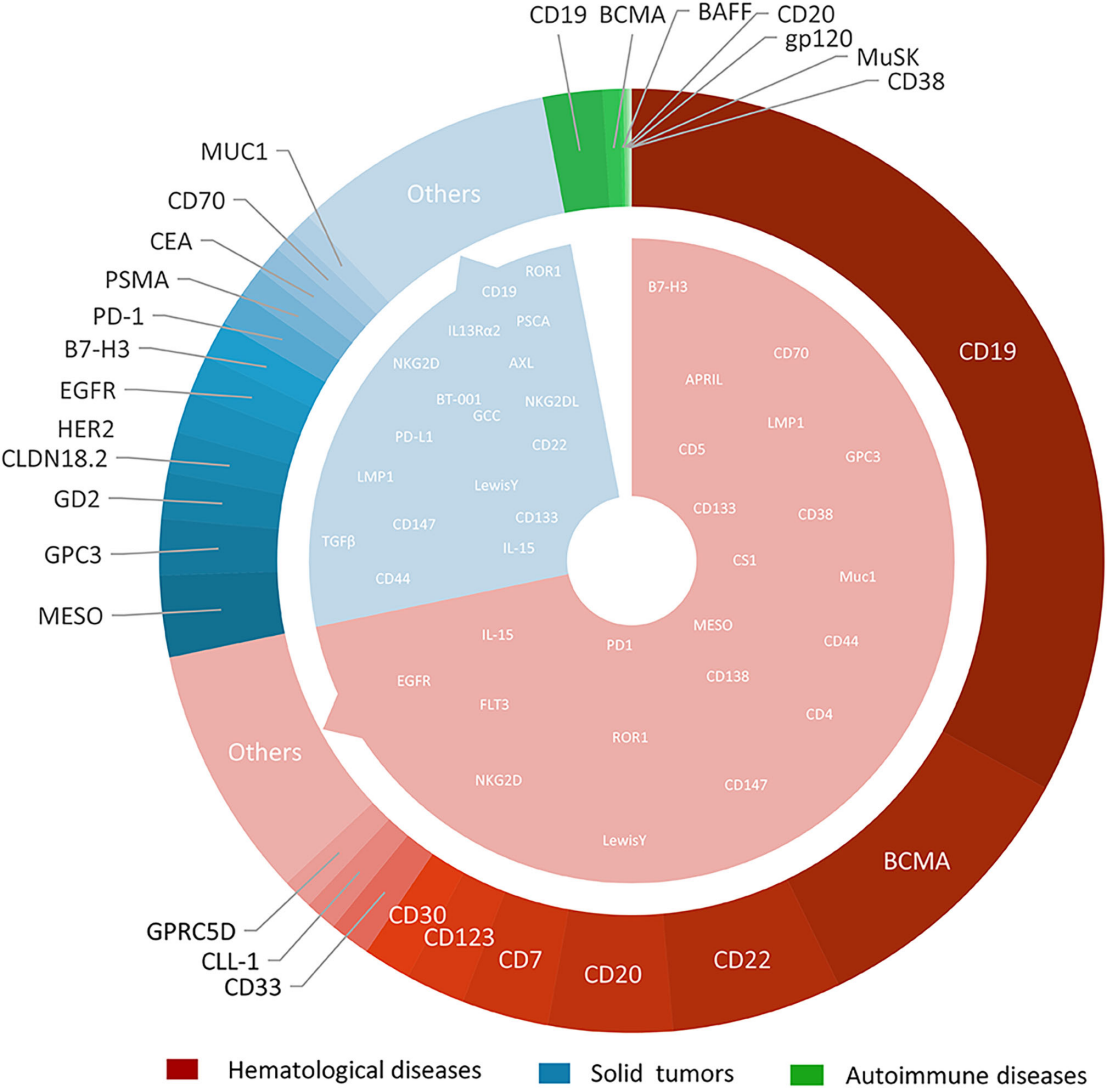
The types and quantities distribution of CAR-T targets in hematological diseases, solid tumors and autoimmune diseases.

Among the 1,580 registered CAR-T clinical trials, 1,457 (92.2%) focused on disease treatment. The majority of these trials targeted hematological malignancies (71.6%), followed by solid tumors (24.6%). Emerging applications include autoimmune diseases (2.75%) and anti-infective or other indications (**Figure 3**).

With the deepening of research and continuous improvement of technology, CAR-T therapy has an increasingly broad application prospect in the field of disease treatment. Both academic institutions and pharmaceutical industry have shown the keen interest in solid tumor CAR- T studies. Compared with 2020, the number of CAR-T studies focus on solid tumor has shown a remarkable increase of up to 170%, far surpassing the 55% growth observed in the field of hematological diseases. The CAR-T trials in solid tumors mainly focus on the liver, gallbladder, and pancreas (14.8%); esophagus, stomach, and colon (12.8%); urinary genitalia (kidneys, bladder, prostate) (7.5%); thyroid, nose and throat, breast (4.8%); and uterus and ovaries (3.9%). The detailed results are shown in **Figure 3**. About 100 trials registered on ClinicalTrials.gov did not target a specific tumor or targeted multiple types of tumors.

It is noteworthy that glioma, a type of nervous system tumor, has gain significant research interest with 37 CAR-T clinical trials registered (25 of which were initiated after 2020).

In autoimmune diseases, trials primarily target refractory systemic lupus erythematosus (SLE), lupus nephritis, systemic sclerosis, refractory Sjogren’s syndrome, myasthenia gravis, neuromyelitis optica spectrum disorders. CAR-T applications also extend to infectious diseases (e.g., AIDS, Epstein-Barr virus) and rare conditions including metabolic disorders and fibrotic diseases.

**Figure 4:**
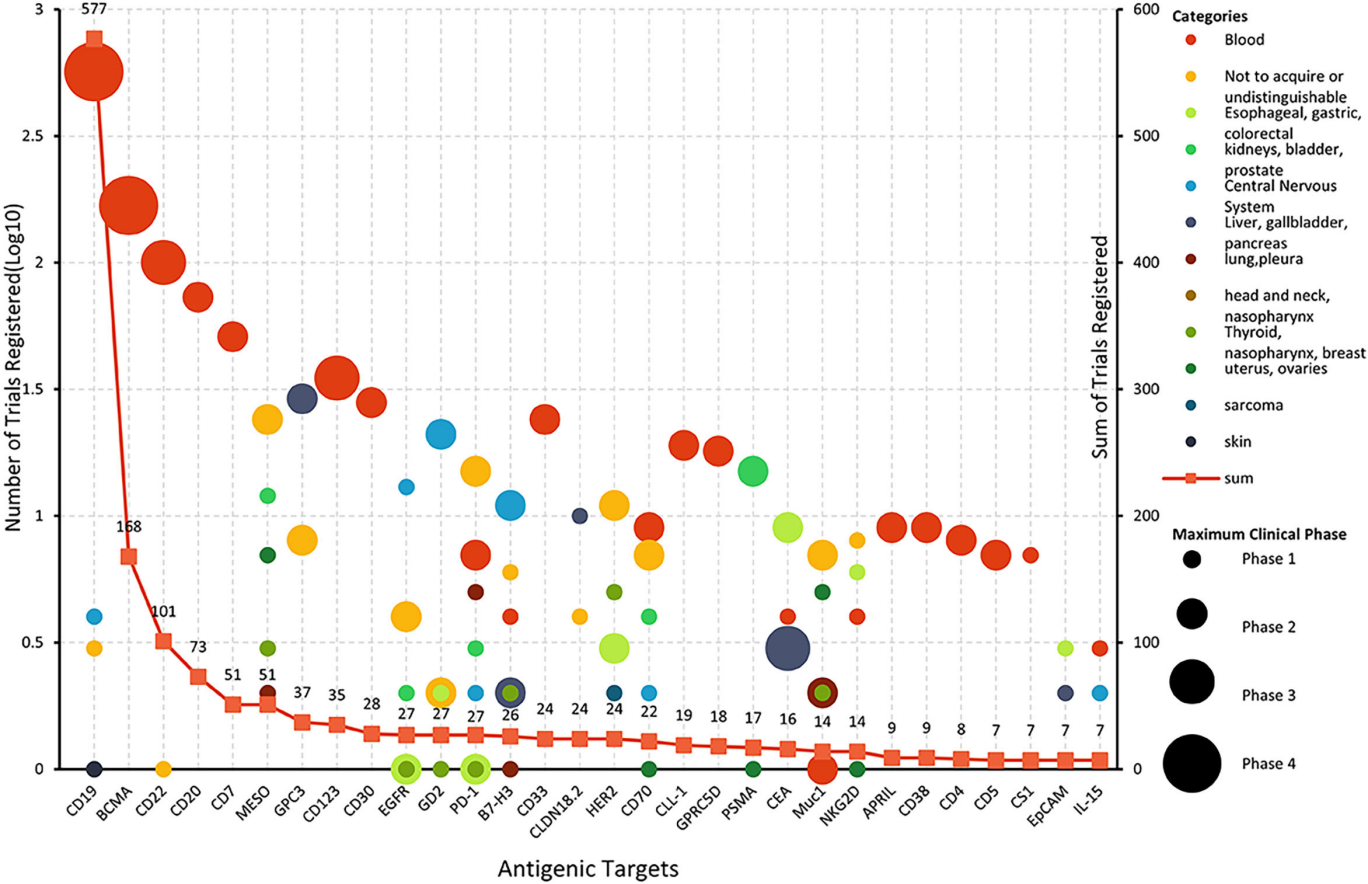
Comprehensive diagram of CAR-T trials targets, indications, quantity, and phase.

The selection of CAR-T targets directly determines the specificity and therapeutic efficacy of CAR-T cells. Up to the search date, 137 antigens had been used as targets for CAR-T clinical trials. Among these, 62 targets were specific to hematological diseases, 90 to solid tumors, and 7 to autoimmune diseases. Notably, 17 targets (including CD19, CD70, B7-H3, etc.) were shared between hematological and solid tumor CAR-T studies (**Figure 4**). 11 targets were identified for CAR-T studies in other disease categories. Overall, the most rapidly expanding targets are in the solid tumors field, with MESO (13.41%), GPC3 (9.22%), GD2 (7.54%), CLDN18.2 (6.70%), and HER2 (6.70%). The most extensively studied targets in hematological diseases remain CD19 (54.41%), B-cell maturation antigen (BCMA) (16.12%), CD22 (9.60%), CD20 (7.01%), and CD7 (4.89%). Multi-target studies are becoming more common with 20.15% in hematological diseases, and 17.60% in solid tumors. We constructed a comprehensive graph (**Figure 4**) to present the target types, quantities, diseases distribution, registration numbers and indications classified by histology of current CAR-T research targets intuitively.

CD19 and BCMA are well-deserved “star targets” in hematological diseases (accounting for 54.36% and 16.11% respectively). BCMA mainly focused on MM (48 of 57 trials), and others focused on B-ALL, lymphoma, and POEMS syndrome. For patients with CD19 CAR-T cell therapy failure, the benefit of existing therapies is limited, and changing the target is a hot spot for researchers to explore.

CD22, a B-lineage-restricted sialic acid-binding adhesion molecule is expressed in nearly all B-cell malignancies. Frank et al. conducted a study involving 41 patients with relapsed or refractory large B-cell lymphoma (R/R LBCL) who received CD22 CAR-T therapy. The study reported an overall response rate (ORR) of 68% and a complete response (CR) rate of 53% in patients with relapsed or CD19-negative LBCL following CD19 CAR-T therapy. The median progression-free survival (PFS) for all patients was 3.0 months and the median overall survival (OS) was 14.1 months (12). Although CD22 has demonstrated efficacy as a target for B-cell acute lymphoblastic leukemia (B-ALL), with over 100 clinical studies conducted either as a single target or in combination with other targets, no therapies targeting CD22 have yet been approved for the treatment of large B-cell lymphoma (LBCL). Meanwhile, CD20, which is overexpressed in more than 90% of B-cell lymphomas, has spurred the initiation of over 70 CAR-T clinical trials worldwide, all in early-phase development.

Owing to lack of tumor-specific T-cell antigens, CAR-T cell fratricide (self-elimination through mutual attack), impaired T-cell regeneration capacity and risk of malignant T-cell contamination in autologous CAR-T products, The development of CAR-T therapy for T-cell malignancies remains largely restricted to preclinical and early-phase clinical trials (13). CD7 is a transmembrane glycoprotein from the Ig superfamily that is commonly expressed on NK cells and T lymphocytes. Research has demonstrated that a significant proportion of T-cell acute lymphoblastic leukemia (T-ALL) and T-cell lymphomas overexpress CD7, rendering it a promising target for the treatment of these malignancies (14, 15). Since CD7 is not only widely expressed on T-ALL cells, but also on the surface of almost 90%-96% of normal T cells, the possibility of CAR-T cell cannibalism should be considered when CD7 is used as a therapeutic target (16).In conjunction with advancements in gene editing technology (e.g., CD7 knockout strategies), CD7 protein blockers, over 70 clinical trials targeting CD7 with CAR-T cells have been initiated.

It is difficult to find specific tumor-associated antigens (TAAs) in solid tumors. Mesothelin (MESO) was highly expressed in a variety of solid tumors, such as pleural mesothelioma, ovarian cancer, and non-small cell lung cancer (NSCLC) and was low expressed in small number of normal tissue cells, such as pleura, peritoneum, and pericardium mesothelin cells, thus posing a good target for CAR-T cell therapy (5). At present, it is the target of the largest number in solid tumors, and 51 MESO target clinical trials have been registered in this study. Glypican-3 (GPC3) is a member of the glypican family of heparan sulfate proteoglycans that plays a critical role in cell differentiation, proliferation, migration, and apoptosis. It is highly expressed in HCC (70%-80%), but hardly expressed in normal tissues, so it is regarded as a “golden” target for hepatocellular carcinoma treatment (17). Claudin 18.2 exhibits broad expression across malignancies, particularly gastrointestinal cancers (e.g., gastric, pancreatic) and other epithelial tumors such as esophageal adenocarcinoma and ovarian carcinoma (18).

## 4 Scientific challenges and translational barriers, emerging strategies and future perspectives

### 4.1 Relapse following CAR-T cell therapy

Drug relapse has emerged as a pervasive and formidable obstacle within the realm of CAR-T cell therapy, significantly impeding therapeutic efficacy and long-term patient outcomes (19). CD19 is extensively expressed in B-cell malignancies and has emerged as the most commonly utilized specific target for hematological malignancies. Despite its widespread application, a significant proportion of patients experience drug-resistant relapses within 12 months following CD19-targeted therapy (demonstrates relapse rates of 30-50% within 12 months post-treatment). This resistance pattern extends to other targets including CD22 and BCMA, with clinical studies reporting comparable relapse rates in BCMA-targeted multiple myeloma therapies (9, 10). The immunological escape of cancer cells through the loss, downregulation, or mutation of cell surface target molecules, coupled with CAR-T cell depletion, constitutes the primary cause of drug resistance relapse. Additionally, the complex microenvironment of solid tumors not only inhibits the activity and function of CAR-T cells but also, in its high tumor load state, exposes CAR-T cells to prolonged high levels of antigen, leading to functional exhaustion (20). Several research directions have been developed to tackle these complex issues (**Table 3**).

**Table 3:** The mechanisms and research directions of relapse following CAR-T cell therapy.

Mechanisms	Directions	Example
Loss, downregulation, or genetic mutation of cell surface target molecules and poor CAR-T cell persistence (CAR-T cell depletion)	CAR structure optimization	Increasing the persistence of antigen recognition, signaling and coupling, joint domains and co-stimulatory domains, or introducing memory-like CARS
	Genetically Edited CAR-T Cells	PD1 coding gene (PDCD1) knockout; TGF-β receptor knockout
	Targeting multiple antigens	CD19/CD20, CD19/CD22, and BCMA/CD38,
Immune regulatory	Combined with immune checkpoint inhibitors (ICIs)	PD1; PD-L1
	Combined with immunomodulatory agents	Lenalidomide
Increase antigen expression or decrease antigen density threshold	Combined with treatments that maintain tumor surface target expression	Gamma-Secretase Inhibitors (GSI) increase the density of BCMA on the surface of malignant plasma cells
	Lowering antigen density threshold	enhancing the affinity of single-chain variable fragments (scFv) for target antigens

#### 4.1.1 CAR structure optimization and editing CAR

Strategies such as augmenting the persistence of antigen recognition, optimizing signaling and coupling domains, refining co-stimulatory domains, and introducing memory-like CARs have been continuously explored and refined (21). We found that more researches used 4-1BB and CD28 as the most commonly costimulatory domains in recent years. The fourth generation of CAR-T cells, also known as T cells Redirected for Universal Cytokine Killing (TRUCKs) or armored CAR-T cells, builds upon the second-generation by incorporating cytokines such as IL-12, IL-15, and IL-18 (22). In our analysis, 36 registered CAR-T studies incorporated relevant cytokines, underscoring the growing interest in this approach. Gene editing technologies, particularly CRISPR/Cas9, have emerged as powerful tools for developing next-generation CAR-T therapies (23). Targeting immunosuppressive pathways has shown promise; for example, knocking out the PD1 coding gene (PDCD1) can mitigate the negative effects of the PD-1/PD-L1 axis on CAR-T cell function (24). TGF-β is the major immunosuppressive regulator within the tumor microenvironment (TME). Studies have demonstrated that knocking out TGF-β signal in CAR-T cells enhances their proliferation and antitumor activity (25). Leveraging the flexibility of CRISPR/Cas9, researchers can simultaneously disrupt single or multiple genes encoding suppressor receptors, effectively “deleting” these receptors from the CAR-T cell surface to improve persistence (26). As of the search date, we searched 14 gene knockout studies have entered clinical trials.

In addition, some experimental therapies are being explored. CAR-CIK cells combine the broad-spectrum cytotoxic activity of cytokine-induced killer (CIK) cells with the antigen-specific targeting of CARs. These constructs are typically engineered using Sleeping Beauty (SB) transposon system, to deliver anti-CD19 CARs (comprising CD28, OX40, and CD3ζ co-stimulatory domains) into donor-derived CIK cells isolated from peripheral blood, thereby generating the CARCIK-CD19 therapeutic product. In a multicenter clinical study (NCT03389035), CARCIK-CD19 cells were used in 13 patients with relapsed B-ALL after HSCT. Complete responses occurred in 61.5% of the patients, as compared with 85.7% of the six patients receiving the two highest doses (27).

#### 4.1.2 Targeting multiple antigens

Targeting multiple antigens can be achieved through several strategies. Dual/multi-targeted CAR-T cells are a combination antigen-targeting design that simultaneously expresses two or more different CAR molecules in the same T cell population (28). Double-signaling CAR-T cells refer to the parallel presentation of two different single-chain antibodies on a single T cell (29). Tandem CAR cells designed with two different targets arranged in tandem on a single T cell can recognize two different antigens in two ways. There are several typical combinations of antigens for dual-target CAR-T therapy: CD19/CD20, CD19/CD22, and BCMA/CD38, other combinations are investigated in clinical trials such as CD19/CD20/CD22, BCMA/CD19 and EGFR/CTLA-4/PD1. Recently, multiple targets, especially dual target CAR-T has been used more and more in clinical practice, about 304 CAR-T clinical trials, 240 of hematologic disease and 64 of solid tumors are registered (among these, 299 were dual-target studies). The disclosed clinical data has shown that dual-target or multi-target CAR-T has great application value. In addition to improving the persistence of response, the dual-target or multi-target CAR-T cell therapy is expected to get the re-generate response in patients after single-target CAR-T treatment recurrent refractory. Shi M et al. developed CAR-T targeting BCMA and CD19 for the treatment of recurrent and refractory multiple myeloma (R/R MM). The ORR was 92%, the median PFS was 19.7 months, and the median overall survival was 19.7 months (30). Researchers of Baylor College of Medicine have developed a novel CAR-T(Smar T) that independently recognizes PSCA and TGF-β and IL-4, delivering independent signals that include: Antigen recognition, co-stimulation and cytokine secretion, when the three key signals are recognized and delivered to T cells, T cells will be activated and expanded at the tumor site, while resisting the inhibition of the tumor environment to ensure their continued long-term survival and effector function (31).

Adaptor CAR-T (AdCAR-T) is a new type of CAR-T technology. Adaptor molecules (such as antibodies or small molecules) are introduced as a “bridge” to connect to a universal CAR at one end and recognize tumor antigens at the other end (32). By replacing different adapters, a variety of antigens (such as CD19, BCMA, solid tumor targets, etc.) can be targeted to solve the problem of single traditional CAR-T target. This multitarget approach not only improves efficacy but also reduces the risk of antigen loss and immune escape (33). Several AdCAR-T products have entered the clinical trial stage. Frigault MJ et al. constructed D-domain adapters for the treatment of MM (target BCMA) (NCT04155749) and AML (target CD123) (NCT05457010). One (1/13) case of grade ≥3 CRS and one (1/13) case of immune effector cell-related neurotoxicity were reported (34). At present, AdCAR-T clinical trials are at the early stage, and the results are still limited, thus there is caution about the prospects of this technology.

#### 4.1.3 Immune regulatory strategies

CAR-T combined with ICIs can effectively enhance the anti-tumor activity of CAR-T therapy by blocking the activation of the “brake” system (35, 36). Our search identified 36 registered CAR-T trials incorporating immune checkpoint inhibitors. PD-1 inhibitors can solve the upregulation of PD-L1 expression caused by tumor microenvironment stress, which is expected to improve the persistence of CAR-T cell therapy and reduce toxic side effects. Chiara Fet al. developed a PD-1 KO CD19-CAR-T that achieved a 68% CR rate in relapsed/refractory B-cell lymphoma without grade > 3 cytokine storm (37).

In a specific study, 33% of patients with B-cell lymphoma received pabolizumab in combination with CD19 CAR-T cell therapy, resulting in an increased CAR-T cell count in peripheral blood (38). Liu et al. constructed a PD1-inhibited anti-CD19 CAR-T that showed 77.8% (7/9) ORR and 55.6% (5/9) CRR in patients with non-Hodgkin lymphoma (NHL), and the CAR-T cells expanded after infusion and remained detectable beyond 12 months in patients with persistent CR (39). CTLA-4, as another immune checkpoint, has been explored as a target in CAR-T therapy in solid tumors. Laboratory studies of the combination of CTLA-4 inhibitors and CAR-T therapy have demonstrated promising prospects (40).

However, no conclusive clinical trial results have been published, due to the adverse effects of double immune activation make it more cautious in clinical studies (41). Lenalidomide is an immunomodulatory drug that exerts direct antitumor effects in multiple myeloma by directly binding to the E3 ubiquitin ligase cereblon and mediating degradation of Ikaros (IKZF1) and Aiolos (IKZF3). A series of studies have demonstrated that the “switch” to control CAR-T activity via lenalidomide is a rapid, reversible, and clinically applicable system (42, 43).

#### 4.1.4 Increase antigen expression or decrease antigen density threshold

CAR-T therapy combined with treatments that maintain tumor surface target expression is also a potential strategy to prevent antigen downregulation. Gama-Secretase inhibitor (GSI) can increase the density of BCMA on the surface of malignant plasma cells and enhance the BCMA CAR-T antitumor activity. Cowan AJ et al. recruited 18 patients with R/R multiple myeloma (MM) to participate in the phase I clinical study treated by GSI combined with BCMA CAR-T. The study firstly clinically demonstrated that GSI reduces soluble BCMA concentration by significantly increasing BCMA surface density on malignant plasma cells. The patients median duration of response was 14.4 months, PFS was 28.8 months, OS was 42 months (44). Novel receptors can lower the antigen density threshold and increase the affinity of scFv to the target antigen. Katsarou et al. engineered a chimeric co-stimulator receptor (CCR) that lacks of the CD3ζ domain. It can activated the prototype CAR at very low antigen density, preventing low antigen escape (45).

### 4.2 Toxicity and safety barriers

On-target off-tumor effects, Immune effector cell associated neurotoxic syndrome (ICANS), and neurotoxicity greatly limits the use of CAR-T cells. Post-translational modification that targets tumors only is a potential strategy to overcome off-target effects such as truncated GalNAca1-O-Ser/Thr and Neuaca2-6-Galnaca1-O-ser/Thr overexpressed in solid tumors. The four main CAR-T cell targets commonly studied are TAG7228, B7-H3, MUC1, and MUC16 (46). In our study, we identified 38 solid tumor clinical trials targeting these antigens. CRS is associated with the production of super physiological cytokines and the proliferation of a large number of *in vivo* T cells, resulting from the release of a large number of cytokines due to the widespread activation of the given CAR-T cells.

IL-1 and IL-6 receptor antagonists have demonstrated efficacy in mitigating CRS (47–49). Interim data from a phase 2 trial (NCT04975555) reported in ASH 2023 showed that early use of siltuximab at CRS grade 1 reduced ICU admissions by 40%, indicating potential for CRS prevention (48). Research indicates that endothelial activation is a pivotal factor in the pathophysiology of ICANS, although the precise etiology of ICANS remains to be fully elucidated. Systemic corticosteroids and antiepileptic drugs are utilized on an as-needed basis to manage these adverse events. Presently, the granulocyte-macrophage colony-stimulating factor (GM-CSF) antibody is under investigation for its potential to ameliorate both ICANS and CRS (50, 51). As of the latest data, only 12% of the clinical trials were designed with more than 5-year follow-up and could not assess late toxicity, and 52 studies registered on ClinicalTrials.gov specifically address adverse reactions associated with CAR-T therapy, that obviously insufficient.

### 4.3 Translational predicament

Despite the significant increase in the number of clinical trials, most of the registered trials remain in early stages with limited patient enrollment, particularly in solid tumors (**Figure 4**). The heterogeneity of solid tumor targets (e.g., MUC1 is expressed in only 60% of breast cancer cases), microenvironment resistance mechanisms, natural physical barriers (e.g., the blood-brain barrier of gliomas), and immunosuppressive factors (TGF-β, PD-L1) lead to the difficulty of CAR-T infiltration, forcing researchers to repeatedly optimize the design of products, which prolongs the early exploration cycle (8). On the other hand, Regulatory agencies such as the FDA and EMA impose stricter on the approval for CAR-T therapies in solid tumors. More comprehensive safety data (e.g. off-target toxicity verification) was demanded, which indirectly pushes up the threshold of phase 3 trials. A more realistic problem is that from laboratory development to preclinical research, and then to clinical trials, CAR-T therapy faces the double challenges of huge research costs and uncertain efficacy. In addition, the cost of individual treatment is extremely high due to individualized preparation, and small and medium-sized enterprises face financing difficulties. Funding shortages are a primary obstacle for CAR-T research, as is fully confirmed in our surveys on the researchers. Insufficient funding appears to be the major reason for the difficulties and termination or withdrawn of CAR-T research, 63.64% (14/22) and 54.55% (12/22) researchers reported respectively (**Supplementary Figure 1**).

Sponsors are not only the source of funding for research implementation, but also a critical bridge between researchers, medical institutions, regulatory authorities and the whole society. Our results show that more than half of the registered CAR-T clinical trials in China are funded by academic institutions, compared to approximately 40% elsewhere. The advantage of academic institutions is that they usually gather a group of excellent professionals, including scientists, clinicians, and technicians, and equipped with advanced experimental facilities and technologies. All these provide robust support for the development of new CAR-T products. In addition, non-profit academic institutions usually focus on social benefits rather than pursuing financial returns, which helps to win trust and support from all sectors of society. Industry funding brings commercial sensitivity and market insights, and is able to quickly capture industry trends and opportunities arising from technological change. and substantial capital, facilitating efficient operations and industrial accumulation. In addition, enterprises often have strong capital strength, efficient operation management mechanism and deep industrial accumulation, and it is easier to obtain financial support from venture capital, private equity financing and other forms.

Mixed-source funding can not only disperse risks effectively, but also promote collaboration between different stakeholders. For example, the close integration of academia and industry enables the rapid translation of the latest basic research results into clinical applications; At the same time, the deep involvement of medical institutions ensures the safety and efficacy of the treatment regimen. Studies have shown that clinical trials with mixed-source funding are more likely to be recognized and supported by regulatory bodies because they typically involve the efforts of multiple stakeholders and reflect broad societal needs (52). Overall, there has been a noticeable increase in the proportion of mixed sources financial support in recent years, with over 30% in our study, and we predicted that it will continue to increase in the future.

### 4.4 Transparency deficits and publication bias

The full disclosure and timely publication of CAR-T clinical research results is the cornerstone to promote scientific progress and clinical translation. Firstly, transparent data sharing can effectively avoid repetitive studies and accelerate the iterative optimization of target selection and engineering strategies. For example, inadequate disclosure of neurotoxicity data in early studies of CD19-targeting CAR-T therapy led to repeated severe ICANS events in multiple subsequent trials, which improved after the ASTCT issued unified management guidelines in 2019 (53). Second, concealment of negative results and failures distorts the evidence chain and bias the meta-analysis - the pooled objective response rate (ORR) of CAR-T in solid tumors was overestimated by 15% because 30% of unpublished negative trials were not included. In addition, inadequate disclosure of results directly threatens patients’ rights.

We conducted a comprehensive search for completed (including terminated and withdrawn) CAR-T cell therapy clinical trials using NCT identifiers on PubMed and Google Scholar, identifying a total of 679 trials. Only 288 trials (18.23%) had published results. Notably, the number of published trials from China was particularly low, with only 111 (14.23%) of the registered studies disclosing results. The publication rate was slightly higher in hematological diseases at 22.12% (211/1042), followed by solid tumors at 17.32% (62/358), while the publication rate for immune diseases was only 15.00%. The low overall publication rate does not preclude the possibility that a small number of studies have not yet been completed. In fact, among the published trials, some are still in the recruitment phase. Industry-sponsored trials tend to delay or selectively release data to protect commercial interests, while academy-led trials often cannot afford the high cost of long-term follow-up due to resource limitations. Our survey on the publish willing supports this fact. Data protection or trade secrets were the primary reasons for non-publication, accounting for 54.55% (12 of 22), followed by negative results or statistically non-significant findings at 22.73% (5 of 22).

Considering the high investment and high-risk characteristics of CAR-T research, it is recommended to promote the construction of public databases like ImmPort, and mandatory sharing of anonymous patient data (such as cytokine levels and genomic integration sites), especially for negative or neutral results. In addition, journal policy reform is recommended: encourage journals to set up “negative results” columns and prioritize review of research results of preregister trials.

In addition, the characteristics of published and unpublished researches found that unpublished studies were more likely to have the smaller enrollment sizes. 68.21% of the unpublished studies had fewer than 50 participants, compared to 82.85% of published studies (P<0.001), and only 1.65% of unpublished studies involved more than 150 participants, compared to 7.69% of published studies (P<0.001) (**Table 4**).

**Table 4:** The finished CAR-T trials registered on ClinicalTrials.gov.

Variable	Published (n=195; N=679)	Not Published (n=484; N=679)	P value
Phases			
Early Phase 1	10 (5.13)	60 (12.40)	0.005
Phase 1	102 (52.31)	215 (44.42)	0.062
Phase 1 and 2	52 (26.67)	130 (26.86)	0.959
Phase 2	20 (10.26)	26 (5.37)	0.022
Phase 2 and 3	1 (0.51)	3 (0.62)	0.869
Phase 3	3 (1.54)	2 (0.41)	0.121
Phase 4	0 (0)	1 (0.21)	0.525
Not application	7 (3.59)	47 (9.71)	0.008
Enrollment			
<50	133 (68.21)	401 (82.85)	<0.001^a^
50-100	38 (19.49)	66 (13.64)	0.056
101-150	9 (4.62)	9 (1.86)	0.043
>150	15 (7.69)	8 (1.65)	<0.001 ^a^
Funding			
Other	92 (47.18)	193 (39.88)	0.081
Industry|Other	43 (22.05)	168 (34.71)	0.001
Industry	37 (18.97)	104 (21.49)	0.465
NIH|Other	8 (4.10)	8 (1.65)	0.057
NIH	10 (5.13)	7 (1.45)	0.006
Industry|Other|NIH	3 (1.54)	2 (0.41)	0.121
Other|Industry|U.S. Fed	2 (1.03)	2 (0.41)	0.345
Start date			
Before 2007	1 (0.51)	2 (0.41)	0.860
2007-2013	11 (5.64)	8 (1.65)	0.004
2014-2020	128 (65.64)	300 (61.98)	0.372
After 2021	55 (28.21)	174 (35.95)	0.053
Number of sites			
Single-center	75 (38.46)	200 (41.32)	0.492
Multiple-center	120 (61.54)	284 (58.68)	0.492

•Data are presented as number (percentage). •^a^Statistically significant.

• Data are presented as number(percentage).

• ^a^ statistically significant;.

## 5 Discussion

CAR-T therapy is currently under investigation for a broad spectrum of diseases, encompassing oncology, autoimmune conditions, infectious diseases, and other areas, thereby revolutionizing the treatment paradigms for several refractory diseases. The sustained increase in registered clinical trials reflects rapid field expansion. According to projections by E. Moreno-Cortes et al., approximately 900 CAR-T clinical trials, excluding those for non-malignant conditions, are anticipated to be registered between 2020 and 2025 (54). Notably, our analysis identified 1,139 CAR-T trials initiated from January 2020 to April 2024, surpassing this projection. China remains the country with the largest number of CAR-T studies, followed by the United States However, growth rates in other regions are particularly striking, with a 422.45% increase in trial numbers over the past five years compared to the early 2020s.

ALL, NHL, MM continue to be the predominant indications for CAR-T therapy. According to the latest data, 12 CAR-T products have been approved in China and the United States, all targeting hematologic malignancies. Other hematological conditions, such as r/r immune thrombocytopenia, sickle cell disease, beta thalassemia, advanced or high-risk myelodysplastic syndrome, relapsed/refractory Langerhans cell histiocytosis, and light chain amyloidosis, are also under investigation, though these remain in early-stage clinical trials.

While the number of CAR-T trials in solid tumors is smaller than in hematologic malignancies, their growth rate has surpassed that of hematologic studies (**Figure 3**). Tumor-associated antigens such as MSLN, EGFR, HER2, cldn18.2, CEA, PSMA, etc., have been studied and reported encouraging results. For example, CT041 (targeting Claudin18.2) developed by Keji Pharmaceutical has shown outstanding efficacy in gastric and pancreatic cancer patients, with an ORR of 48.6% and a CR rate of 73% (18). Similarly, P-PSMA-101 CAR-T cells exhibited significant anti-tumor activity in Phase 1 trials for metastatic castration-resistant prostate cancer (mCRPC) (55).

Despite these promising results, CAR-T cell therapy remains associated with safety issues such as CRS, CAR-T-associated encephalopathy and secondary cancer risk (56). Several innovative strategies are being explored to enhance the efficacy and safety of CAR-T therapy. Logic-gated CAR design: utilizing tumor-specific antigen loss as activation triggers to precisely target malignant cells (57). Hypoxia-responsive CAR-T cells: engineering T cells to detect and adapt to tumor microenvironment hypoxia while enhancing antigen recognition specificity. Advanced delivery systems: developing nanocarrier-based platforms to improve CAR-T cell migration, tumor infiltration, and persistence. In general, CAR-T applications in solid tumors are predominantly in early-phase clinical trials, and their therapeutic potential requires further validation through expanded clinical datasets (58). Moreover, current limitations are compounded by incomplete disclosure of trial outcomes.

Beyond the scientific challenges, the advancement of CAR-T therapy requires coordinated efforts across regulatory frameworks, clinical trial design, and cost-effectiveness optimization.

The current CAR-T trials predominantly remain small-scale and single-center studies with inadequate long-term follow-up. Standardized trial implementation is critical for validating the safety and efficacy in participants. While regulatory agencies are intensifying oversight of CAR-T research processes, dynamic adjustments will be essential as technological advancements and clinical experience accumulate.

Another pressing challenge lies in enhancing patient accessibility through cost reduction and real-world evidence generation. Although recent studies have initiated cost-effectiveness analyses (59, 60), comprehensive solutions must address manufacturing expenses and reimbursement policies. In China, for instance, less than 15% of CAR-T therapies are covered by medical insurance, with off-label use persisting in clinical practice. Key strategies for affordability include universal CAR-T platforms, non-viral vector optimization (e.g., establishing consensus guidelines for Sleeping Beauty transposon systems), and policy incentives.

Despite persistent challenges, CAR-T therapy continues to attract robust multi-sector engagement, reflecting strong confidence in its clinical potential. Survey data from researchers (n=81.8% valid responses) revealed 86.4% prioritize alliance-building and multi-institutional collaboration, while 68.2% identified cost reduction and indication expansion as critical development priorities. Governmental policy reinforcement and funding allocation were endorsed by 59.1% as key accelerators.

Although limited by suboptimal questionnaire retrieval rates (attributable to outdated contact information and geographic dispersion), these stakeholder insights remain strategically valuable. We advocate systematic integration of researcher, patient, and policymaker perspectives to inform CAR-T development roadmaps, particularly in addressing real-world accessibility barriers.
