# Supplementary material for: CAR-T cell therapy clinical trials: global progress, challenges, and future directions from ClinicalTrials.gov insights
Source: Front Immunol. 2025 May 20;16:1583116. doi: 10.3389/fimmu.2025.1583116 (PMC12129935; doi:10.3389/fimmu.2025.1583116)
Supplement: Supplementary file 1 [file DataSheet1.docx]

**Supplementary Fig 1. Survey on CAR T research situation**

**Q1. Reasons for termination or withdrawn of previous CART studies you hosted/participated in.（check all that apply）**

-Not Applicable （The research hosted/participated does not involve termination or withdrawn）

-Insufficient funds

-Security issues

-Poor expected efficacy

-Difficulty recruiting

-Too many similar competitive products, strategic adjustment

-The supply of experimental CAR T is at risk

-The cost is too high and the expected benefits are out of balance

-Passive suspension (ethical considerations, or regulatory requirements)

-Other (please specify)

**Q2. Difficulties in CAR T researches that you hosted/participated are（check all that apply）**

-Insufficient funds

-Low success rate

-High security risk

-Difficulty recruiting subjects

-Similar CAR T products are updated too quickly, Loss of competitiveness

-Multi-center collaboration is difficult

-Standardization Implementation

-Other (please specify)

**Q3. Do you intend to publish the research results?**

-Published already

-Yes

-No

**Q4. Reasons why results are not published or are not intended to be published. （check all that apply）**

-Published already

-The results were negative or not significant statistically

-Data protection or business secrets

-Loss of interest

-Lack of funding

-Lack of time

-Other (please specify)

**Q5. What direction do you think the future CAR T research should focus on? （check all that apply）**

-Expansion of indications

-Associated adverse events

-New targets

-Unknown disease mechanism

-Mechanism of drug resistance and relapse

-New Manufacturing technology

-Optimization of structural design

-Real-world research on products already on the market

-Reduce Product cost

-Combination therapy

-Other (please specify)

**Q6. Measures that you think can improve the current status of CAR T research include（check all that apply）**

-Clarify and standardize the implementation process

-The government increased policy and financial support

-Government and industry cooperation

-Establish the database of subjects (especially in areas with relatively low medical resources)

-Establish alliances and strengthen multi-regional cooperation

-Timely sharing of research information

-Other (please specify)

**Q7. Your opinion on the future of CAR T research**

-Better

-About the same

-Worse
